# Player Sex and Playing Surface Are Individual Predictors of Injuries in Professional Soccer Players

**DOI:** 10.3390/pathophysiology29040048

**Published:** 2022-10-26

**Authors:** Zakariya H. Nawasreh, Mohammad A. Yabroudi, Ahmad A. Darwish, Wesam A. Debes, Khaldoon M. Bashaireh

**Affiliations:** 1 Division of Physical Therapy, Department of Rehabilitation Sciences, Jordan University of Science and Technology, P.O. Box 3030, Irbid 22110, Jordan; 2 Department of Special Surgery, College of Medicine, King Abdullah University Hospital, Jordan University of Science and Technology, P.O. Box 63001, Irbid 22110, Jordan

**Keywords:** football, risk factors, soccer-related injury, sport injury, synthetic surfaces

## Abstract

Background: The factors contributing to soccer injuries and their influence on the occurrence of injury are controversial and inconclusive. This study aimed to determine the association between player characteristics and playing factors with injuries in professional soccer players. Methods: One hundred and fifty-two professional soccer players completed a self-administered questionnaire that asked about demographic information and injury profile, the type of playing surface on which they sustained their injury, medical treatment, and the time lost due to soccer injury at the end of the soccer season. Results: The injury rate was 44.74% (*n* = 68; males: 61.50% (*n* = 56), females: 19.70% (*n* = 12)). Players’ age (OR: 1.15, 95%CI: 1.05–1.25, *p* < 0.002) and BMI (OR: 1.21, 95%CI: 1.06–1.38, *p* < 0.003) were significantly associated with soccer injuries. After adjusting for age and BMI, players’ sex (OR: 5.39, 95%CI: 2.11–13.75, *p* < 0.001), previous soccer injury (OR: 3.308, 95%CI: 2.307–29.920, *p* < 0.001), and playing surfaces (OR: 11.07, 95%CI: 4.53–27.03, *p* < 0.001) were the significant predictors of soccer injuries. Conclusion: Players’ age, BMI, sex, previous soccer injury, and playing surface were associated with injuries among professional soccer players. Old male athletes with high BMI, previous soccer injuries, and playing on natural grass were more likely to sustain soccer injuries than young female players with low BMI who had no previous injuries and played on synthetic surfaces.

## 1. Introduction

Soccer is one of the most commonly played sports in the world, it was estimated that 265 million athletes play soccer globally [1]. In Jordan, soccer is considered the most popular sport. In 2018–2019, there were 12 Premier League teams, 14 Division I teams, and 12 Division II or lower registered teams in the Jordanian football association [2]. However, the injury rate among professional soccer players in Jordan remains unknown. Furthermore, the intrinsic and extrinsic factors associated with the occurrence of injuries among professional Jordanian soccer players are unknown.

The injury rate among professional soccer players is high and varies according to severity [3,4,5,6,7]. Among European professional male soccer teams, it is 8.0 per 1000 player-hours, which is equivalent to two injuries for each player in a soccer team per competitive season [5]. The high rate of injuries is also well-documented in youth and adult players [8], with most of the injuries sustained in the lower extremities and during the game, as opposed to practice [3]. Soccer is a very demanding activity that includes sprinting, jumping, landing, cutting, pivoting maneuvers, and overuse. Importantly, it involves frequent physical contact between players, which further exacerbates the risk of injury [3]. Incurring high-grade injuries in soccer is devastating for players, given the wide range of structural damage and psychological frustration. Soccer injuries can be mild, moderate, or severe injuries, with moderate and severe injuries usually associated with restricting participation in competitive games, lower performance, and increased risk for the second injury, and subsequently, they may affect the player’s career [5]. Furthermore, moderate and severe soccer injuries are usually economically burdened due to medical treatments, players’ compensations, and human resources. To develop effective preventive strategies, it is essential to characterize the incidence, severity, and nature of injuries among professional soccer players. Additionally, it is necessary to identify the risk factors associated with soccer injuries in professional teams to develop tailored prevention strategies that can address these factors.

Several studies have reported soccer injury profiles, including the prevalence, type, severity, location, injured tissue, and injury patterns, in youth and adult players in different countries [9,10]. These characteristics seem to be affected by multiple intrinsic and extrinsic factors, including age, sex, player position, professional status, team division, playing surface, and history of previous injuries [11,12,13,14]. Player sex may play a role in injury incidence because of the difference between males and females in the nature and demand of sports, the level of sport participation, physiological factors, and psychological factors [15,16,17]. Player age may also affect soccer injuries [18], with an increased injury rate among older players suggested to be related to physiological changes with increased age [19]. Furthermore, there are differences in the participation level (number of soccer games and training), physical and psychological loading, and the time of recovery between teams playing at different levels of soccer competition [8]. However, there are inconsistencies in the reported injury incidence, pattern of injury, and traumatology between players participating in different levels of soccer competition [14,20].

Other factors that may influence the occurrence of soccer injuries include a previous injury [13,14], player position [10,21], and playing surfaces [22,23,24]. Players with a history of previous injuries are at a high risk of sustaining further injuries [13,14]. Having a previous injury has been suggested to result in impairments and functional limitations that place players at heightened risk of sustaining further injury [25,26]. The player position has also been suggested as a risk factor for soccer injuries, as each position requires different physical demands, tackling the players of opposing teams, and the technical skills and maneuvers specific for each position in the soccer team [10,21]. Therefore, players in each position are prone to sustain different types of soccer injuries. Different playing surfaces (artificially synthesized and natural grass) have been suggested to exert different effects on the rates and types of soccer injuries [22,23,24]. Although there are disparities in the incidence rates, types, and severity of soccer injuries when stratified by these risk factors, the influences and contributions of these factors to the occurrence of injuries in professional soccer players have not been well-studied. Additionally, few studies have investigated the influence of individual and combined effects of the intrinsic and extrinsic factors related to player characteristics in predicting the occurrence of injuries in professional soccer players. Therefore, the purpose of this study was to determine the injury rate and the association between players’ characteristics (age, sex) and playing factors (team division: Premier League or Division I, playing surface, and players’ position) with injuries in professional soccer players. It was hypothesized that older male players playing in highly professional teams and on natural grass are associated with soccer injuries.

## 2. Materials and Methods

Professional soccer teams registered by the Jordanian Football Association were contacted to participate in this study. The teams’ coaches were contacted via phone calls regarding study participation, and appointments were set for personal visits. During the personal visit, there was a discussion about the nature and importance of conducting this study. Professional soccer players in the Premier League and Division I teams, from all positions, aged 16–35 years, and who played with their teams for more than six months were eligible to participate in this study. The soccer players who had injuries outside soccer playing (practice or competitive game), had no team during the last two years, did not play for their team due to retirement, or had other medical conditions or diseases (i.e., cardiopulmonary, or neurological diseases) that affected their ability to play soccer were excluded. To maximize the generalizability of the study results, players’ recruitment was not restricted to a specific region of the country. The study was conducted per the Declaration of Helsinki, and the study protocol was approved by the Institutional Review Board of Jordan University of Science and Technology and King Abdullah University Hospital. All soccer players signed an informed consent form before participating in this study. For those participants between 16 and 18 years old, permission from the child’s parent or legal guardian was sought and documented on the informed consent form.

### 2.1. Data Collection

The soccer players’ demographic data (age, sex, weight, height, team division (Premier League and Division I), time joining the current team, and position) were provided by the coaching/training staff of their teams. Furthermore, they provided information about the type of playing surfaces and the number of practice sessions and games played on each surface. The coaching/training staff indicated the generation of synthetic surfaces. The medical records of soccer players’ injury profiles during the last two years (i.e., whether the injury occurred during soccer practice or games or non-soccer participation, injury location, injured body part, mechanism of injury, type of injury, name of the injured ligament and muscles, injury status (partial (grades I and II) or complete injuries (grade III), and the playing surface on which they sustained their injuries) were provided by the teams’ medical staff (physicians, athletic trainers, physical therapists). Furthermore, the teams’ medical staff indicated whether the soccer players received medical treatment due to their soccer injury, whether the soccer injury limited their ability to participate in soccer practice or games, and the duration they were not able to participate in soccer practice or competition games due to the injury.

### 2.2. Injury Definition

Injury occurrence was defined as any physical damage resulting from soccer participation in practice or competitive games that required medical attention and resulted in a player being unable to take full part in future training or game following the injury [27]. The soccer players were classified based on the injury definition into injured (injured group) or non-injured (non-injured group). Medical attention was defined as players seeking medical treatment by seeing a physician or any of the medical teams (i.e., physical therapist) or requiring surgical management due to the injury. The injury rate per 10,000 player-day was calculated as the number of injuries divided by the total players’ exposure time × 10,000, with players’ exposure time calculated as the total number of hours of soccer participation (practice and game) for the players. Further, the injury rate per 10,000 player-day for each playing surface was calculated as the number of injuries on each playing surface divided by total exposure of playing surfaces × 10,000.

### 2.3. Independent Measures

Age: The players’ age in years was used as a continuous measure;

BMI: The players’ BMI was computed and used as a continuous measure;

Sex: The soccer players were dichotomized based on their sex (male, female);

Dominant lower extremity: The soccer players were dichotomized based on the dominant lower extremity (right, left);

Team division: The soccer players were dichotomized based on their team division (Premier League and Division I);

Previous soccer injuries: The players were dichotomized based on whether they had sustained a previous soccer injury (yes, no);

Playing surface: The soccer players were grouped based on the playing surface (natural grass or synthetic surface) that they sustained their injuries while playing soccer;

Player position: The soccer players were grouped based on their position, namely goalkeeper, defense, side, middle, and anterior/strikers.

### 2.4. Statistical Analysis

An independent *t*-test was used to determine the significant differences in age and BMI between the injured and non-injured groups. A chi-square test was used to determine the significant differences between the groups relative to the dichotomized variables (sex and team division). Univariate logistic regression analysis was used to determine the association between player characteristics, playing factors, and soccer injuries (R^2^: Nagelkerke R square, odds ratio (OR), with 95% confidence interval (95%CI)). If the demographic measures were significantly different between the injured and non-injured players, the significant measures were adjusted for in the univariate and multivariate models. The data from both groups were used for logistic regression analysis. The β coefficients were reported to indicate the regression relationship between the independent measures and soccer injuries, and an alpha level of <0.05 was considered statistically significant (R^2^: Nagelkerke R square, odds ratios, and 95% confidence interval (95%CI)). The assumptions for no multicollinearity (VIF < 10) and no outliers or influential cases (Leverage and Cook) were met. All statistical analyses were performed using SPSS software (version 25.0; IBM Corp, Armonk, NY, USA).

## 3. Results

### 3.1. Soccer Injury Prevalence

In total, 152 soccer players (91 males, 61 females) from 12 Division I and II teams participated in this study. Sixty-eight of the players sustained soccer injury (44.73%; 95%CI, 36.7–53%, males: 61.50% (*n* = 56), females: 19.70% (*n* = 12)), and the overall rate of injury per 10,000 h of playing was 59.86 (males: 49.30, females:10.56). The total exposure time was 69,291 h (61,518 practice hours and 7773 competitive game hours). The mean practice exposure and competitive game exposure per player were 283.59 ± 46.25 h and 35.94 ± 12.6, respectively, during the season. The synthetic playing surface used in this study was a third- and fourth-generation artificial turf.

There were significant differences in the players’ age, BMI, and sex between the injured and non-injured players (*p* ≥ 0.003), while no differences were observed in exposure between the groups (*p* = 0.834) (Table 1). There were significantly more male players (*n* = 56) who had injuries than injured female players (*n* = 12) (*p* < 0.001).

### 3.2. Types and Locations of Soccer Injuries

There were 41 ligament injuries, of which 75.6% (*n* = 31) were partial tears (grade I or II), and 24.4% (*n* = 10) were complete ligament tears (grade III), with the ankle ligament and anterior cruciate ligament tear being the most injured ligaments. Furthermore, there were 35 muscle injuries, of which 91.4% (*n* = 32) had partial muscle injury (grade I or II), and 8.6% (*n* = 3) had complete muscle injury (grade III), with the hamstring being the most injured muscle, followed by the hip adductor muscles and shoulder and arm muscles. Eight players sustained an injury to the meniscus, and there were sixteen other injuries, including contusions and fractures (Table 2).

### 3.3. Univariate Logistic Regression

Player’s age (R^2^: 10.2%, β: 0.137, OR: 1.15, 95%CI: 1.05–1.25, *p* < 0.002) and BMI (R^2^: 7.8%, β: 1.19, OR: 1.21, 95%CI: 1.06–1.38, *p* < 0.003) had significant associations with soccer injuries. After adjusting for player age and BMI, the sex of the soccer players had a significant association with soccer injuries (R^2^: 25.3%, β: 1.68, OR: 5.39, 95%CI: 2.11–13.75, *p* < 0.001), and the injury rate for male players (61.50% (*n* = 56)) was significantly higher than for females (19.70% (*n* = 12)) (*p* < 0.001). Having a previous soccer injury also had a significant association with soccer injury (R^2^: 20.1%, β: 2.12, OR: 3.308, 95%CI: 2.307–29.920, *p* < 0.001), and the injury rate for players with a previous injury was 84.2%, which was significantly higher than those who had no previous injury (39.1%, *p* < 0.001). Furthermore, the playing surface had a significant association with soccer injury (R^2^: 34.6%, *p* < 0.001), and the rate of injury on synthetic surfaces (13.95% (*n* = 6)) was significantly lower than on natural grass (56.88% (*n* = 62)) (β: 2.40, OR: 11.07, 95%CI: 4.53–27.03, *p* < 0.001). The total number of playing hours on synthetic surfaces was 24,252 h (game: 2720 h, practice: 21,532 h) and on natural grass was 45,039 h (game: 5053 h, practical: 39,986 h). The rate of injury per 10,000 h for the synthetic surfaces was 2.47 and 13.77 for natural grass. The synthetic surfaces used third-generation (65%) and fourth-generation (35%) technologies. The dominant side, team division, and player position were not significantly associated with soccer injuries (*p* > 0.05) (Table 3).

## 4. Discussion

The rate of soccer injuries that required medical attention leading to time loss from soccer participation was 45%, and the injury rate per 10,000 h of playing was 59.86. Ligaments were the most common injured structure among professional soccer players, followed by muscle injuries to the hamstring, hip adductors, and shoulder and arm muscle groups. The ankle and the knee were the most common joints that sustained ligamentous injuries. The players’ age, BMI, sex, previous soccer injury, and playing surfaces were associated with injuries among professional soccer teams. These findings support the hypothesis of this study, in which old male players who had high BMI, previous injury, and playing on natural grass surfaces were at high risk for sustaining soccer injuries, compared with young, female players who had low BMI, no history of previous soccer injuries, and played on synthetic grass. The findings of this study may help determine the factors associated with soccer injuries among professional players and to identify the players who are at risk of sustaining soccer injuries. Furthermore, the findings of this study may provide advice in attempting to reduce the risk of sustaining soccer injuries, in which older male players with a high BMI might reduce their weight, and synthetic surfaces might be used as playing surfaces for professional soccer games to lower the risk of sustaining soccer injuries.

The injury rate among professional soccer players in our study was higher than that reported by others [28,29,30]; however, it is consistent with recently reported rates [28,30]. A study by Morgan and Oberlander reported that 64% of Major League soccer players sustained an injury [31]. In the Netherlands, 62.7% of male professional soccer players sustained injuries [32]. Furthermore, the injury rate in this study is within the rates reported by international tournaments such as World Cup and British League (44 to 81 injuries per 1000 exposure hours) [28,30]. The variation in the injury rates between this study and those studies that have previously reported different injury rates of soccer injuries might be due to the differences in the methods used to compute the injury rate, including players with different professional levels and ages and variations in the time duration of monitoring the injury rate. Furthermore, differences in the nature, importance, and intensity of competition may account for the differences in injury rates [33].

The current study revealed that players’ age and BMI were associated with soccer injuries among professional soccer players. In this study, old players were 1.15 times more likely to sustain soccer injuries than young players. This finding is consistent with several previous studies reporting that the rate of soccer injuries is higher in older players who played for youth and adult soccer teams [10,19,34]. It was previously reported that the player’s age is associated with both the number and severity of muscle and tendon injuries [10]. It has been suggested that older players sustain a higher rate of injury owing to physiological changes [19]. This was advocated because the muscle mass, the cross-sectional area, and the tendon’s ability to store and utilize kinetic energy and transmit forces decrease with age [19]. Furthermore, older players could play soccer more in terms of frequency, load, and intensity, and sustained prior injuries that may have placed them at a higher risk for injuries than younger players [25]. For the BMI, players with high BMI were 1.21 times more likely to sustain soccer injuries than those with low BMI. Previous studies have demonstrated that players with high BMI are at an increased risk for sustaining sport-related injuries in the lower extremities [35]. Players with higher BMI are also at higher risk of sustaining injuries that cause long time loss from participation in sports [35]. This could be because players who have a higher BMI tend to sustain sport injuries owing to the high force applied to the joints of the lower extremity and the surrounding soft tissues.

Players’ sex also had a significant association with soccer injuries, with male players being 5.39 times more likely to sustain a soccer injury than female players. Several studies have reported similar results to this study [36,37]. This may have resulted from more participation in competitive games and training hours among male players than females. However, a study by the National Collegiate Athletic Association reported no difference between male and female soccer players in terms of overall injury [38]. Therefore, the influence of sex on soccer injury is still controversial. Future studies may need to control for the frequencies, load, intensity, and duration of playing soccer while determining the incidence of injury and the influence of sex on injuries in professional soccer players.

Playing surfaces had an association with soccer injuries and explained one-third of the soccer injury variance, with the rate of injury per 10,000 player-day being 1.94 for synthetic surfaces and 16.10 for natural grass. In this study, the athletes playing on natural grass were 11 times more likely to sustain soccer injuries than those playing on synthetic surfaces. It was found that collegiate male soccer players who played on natural grass had higher injury rates and lower rates of serious injuries than those playing on synthetic surfaces [8,39]. The synthetic surface was suggested to be more favored than natural grass, even under severe weather conditions (cold, wet, and hot weather) [8]. Other studies have reported that natural grass was not different from synthetic surfaces in acute soccer injuries in either practice or competitive games [23,40]. However, playing on synthetic surfaces has been shown to result in a higher incidence of low-back pain in adolescent soccer players than in those playing on natural grass [41]. Furthermore, the injury rates of the anterior cruciate ligament rupture [24] and ankle sprains [24] were higher when playing on synthetic surfaces than on natural grass in American football league games. This could be because natural grass has different characteristics in terms of shoe-field traction [42], inappropriate friction between shoe fields, stiffness, and disproportionate growth rate, which makes it uneven compared with synthetic surfaces [43]. The high rate of soccer injuries sustained on natural grass in this study might be because of the high number of natural grass playing fields in the country compared with synthetic surfaces. Furthermore, the currently used synthetic surfaces are third- and fourth-generation synthetic surfaces. These generations were developed to optimize and enhance sport participation and injury prevention, which may have contributed to the lower injury rate on synthetic surfaces than on natural grass. In this study, some factors were not controlled for, including age, quality, maintenance of the playing surface, and shoe types and styles. Therefore, future studies should consider controlling for these factors to determine the association between the playing surfaces and soccer injuries.

The findings of this study also revealed that players with a previous soccer injury had an association with sustaining soccer injuries among professional players and explained 20.1% of the variance. The soccer players who had previous soccer injuries were 3.3 times more likely to sustain a soccer injury than those players who did not have a previous soccer injury. This finding is in agreement with the studies of Ekstrand and Gillquist [13] and Nielsen and Yde [14], who found that, in specific body parts (ankle, knee, and groin), having a previous injury was a significant factor influencing the occurrence of secondary injuries. Our data also agree with those of Ekstrand and Gillquist [13,44], who found that one-third of the injuries in athletes with previous injuries were followed by second injuries. It was advocated that having a previous injury may cause biomechanical and flexibility changes, proprioceptive deficits, range of motion deficit, muscle weakness, and scar tissue accumulation that, in turn, may lead to incurring secondary injury [25,26]. Furthermore, there might be negative impacts on players’ performance, especially in those cases in which a player may have not fully recovered or did not receive sufficient rehabilitation to ensure the resolution of symptoms, impairment, and functional deficits developed by previous injuries.

The dominant limb, professional status, and player’s positions were not significant predictors for soccer injuries in this study. The dominant limb did not associate with soccer injuries, which might be because players utilize both limbs while playing soccer. The non-dominant limb is generally used as a stance limb during landing and to bear the body weight, while the dominant limb is generally used for kicking and controlling the ball, which would supposedly place both limbs at risk for injury [45]. Previously, it was reported that injury rate, pattern, and traumatology varied across soccer professional statuses [14]; however, it was not associated with soccer injuries in this study. The influence of professional status on soccer injuries is inconsistent [20], as the professional status of soccer teams varies from season to season based on team performance [12]. In this study, it could be that a group of soccer teams was ranked among different professional statuses during the past few years, which may have led to an increase in the findings of this study. Player positions have also been reported to influence the type and severity of soccer injuries [10,12]. This might be due to the differences in the physical demands, tackling with players of opposing teams, and the technical skills and manoeuvers required for each position [10].

The results of this study should be interpreted in light of these limitations. The cross-sectional design of this study had a recall bias, which may have affected the study findings. In this study, only professional soccer players between 16 and 35 years old and who played for high-level status were included. Therefore, the findings of this study cannot be generalized to soccer players who are younger than 16 years or older than 35 years, or those who play in lower divisions (II, III, or IV). Further, there was a large difference in the practice hours on the different playing surfaces, which could affect the result of the prediction model. Other limitations attributed to the division of training, coaching, and promotions for each team, the number of times and the time duration for each team to stay in the same division, and the internal and external workload variables that were not controlled in the current study. Therefore, future research is necessary to control for these variables to better detect the influence of players’ characteristics and playing factors and their association with injuries in professional soccer players.

## 5. Conclusions

In this study, almost one-half of the players sustained a soccer injury that required medical treatment and restricted their soccer participation. The measures related to players’ age, BMI, sex, previous soccer injury, and playing surface were associated with sustaining soccer injuries among professional soccer players. Old male players with high BMI, previous soccer injuries, and playing on natural grass surfaces were more likely to sustain soccer injuries than young female players with low BMI, no previous soccer injuries, and playing on synthetic surfaces. The findings of this study may help determine the factors associated with soccer injuries among professional players. The influence of the player’s characteristics, the history of previous soccer injury, and playing surfaces should be taken into consideration, as they may help identify those players who have a risk for soccer injuries. Older male players with high BMI might be advised to reduce their weight to lower the risk of sustaining soccer injuries. Furthermore, synthetic surfaces might be used as playing surfaces for professional soccer games to also help reduce the risk of sustaining soccer injuries.

## Figures and Tables

**Table 1:** Demographic information, the time between injury and study participation, and exposure for injured and non-injured soccer players (mean (SD)).

	Injured (*n* = 68)	Non-Injured (*n* = 84)	*p*-Value
Sex (male/female)	(56/12)	(35/49)	<0.001
Age (years)	23.13 (3.89)	20.68 (4.62)	0.001
BMI (kg/m^2^)	22.94 (2.58	21.62 (2.73)	0.003
Time between injury and study participation (months)	18.49 (11.4)	_______	______
Exposure (hour)	1207.55 (388.72)	1114 (368.18)	0.834

**Table 2:** Number, type, and name of injured structure stratified by players’ sex, team division, playing surface, and players’ positions (number of injured athletes).

		Sex (Male (*n*))	Team Division (Premier League (*n*))	Playing Surface (Natural Grass (*n*))	Player’s Positions				
					Striker (*n*)	Defense (*n*)	Goalkeeper (*n*)	Midfield (*n*)	Side (*n*)
Partial or complete ligament injury	Partial tear (total = 31)								
	Wrist ligament (*n* = 1)	(1)	(0)	(1)	(0)	(0)	(1)	(0)	(0)
	Lateral ankle ligament (*n* = 9)	(5)	(4)	(7)	(2)	(3)	(0)	(2)	(2)
	Medial and lateral ankle ligaments (*n* = 7)	(5)	(4)	(6)	(0)	(1)	(2)	(3)	(1)
	Medial ankle ligaments (*n* = 6)	(4)	(1)	(3)	(1)	(1)	(1)	(3)	(0)
	Medial collateral ligament (*n* = 2)	(1)	(1)	(1)	(1)	(0)	(0)	(0)	(1)
	Lateral collateral ligament (*n* = 2)	(2)	(1)	(2)	(0)	(2)	(0)	(0)	(0)
	Anterior cruciate ligament (*n* = 4)	(3)	(4)	(4)	(0)	(2)	(1)	(1)	(0)
	Complete tear (total = 10)								
	Lateral ankle ligament (*n* = 1)	(1)	(1)	(1)	(0)	(1)	(0)	(0)	(0)
	Medial ankle ligament (*n* = 1)	(1)	(0)	(1)	(0)	(0)	(0)	(1)	(0)
	Anterior cruciate ligament (*n* = 8)	(7)	(4)	(8)	(0)	(4)	(0)	(3)	(1)
Muscle injury status (partial or complete rupture)	Partial muscle injury (total = 32)								
	Arm and shoulder muscles (*n* = 6)	(4)	(4)	(4)	(1)	(1)	(1)	(2)	(1)
	Lower back muscles (*n* = 2)	(2)	(2)	(2)	(0)	(1)	(1)	(0)	(0)
	Hand and wrist muscle (*n* = 2)	(1)	(1)	(1)	(0)	(1)	(0)	(0)	(1)
	Hip Adductor muscle (*n* = 5)	(4)	(3)	(5)	(1)	(2)	(0)	(2)	(0)
	Anterior thigh muscle (Quadriceps) (*n* = 3)	(2)	(2)	(1)	(0)	(2)	(0)	(0)	(1)
	Hamstring muscle (*n* = 11)	(9)	(7)	(10)	(2)	(4)	(0)	(3)	(2)
	Leg muscle (*n* = 3)	(1)	(2)	(1)	(1)	(1)	(0)	(0)	(1)
	Complete muscle injury (total = 3)								
	Hip Adductor muscle (*n* = 1)	(1)	(0)	(1)	(0)	(0)	(0)	(0)	(1)
	Hamstring muscle (*n* = 2)	(1)	(0)	(1)	(0)	(0)	(0)	(1)	(1)
Meniscus injury	Medial meniscus (*n* = 3)	(2)	(1)	(2)	(1)	(1)	(0)	(1)	(0)
	Lateral meniscus (*n* = 5)	(4)	(3)	(3)	(2)	(1)	(0)	(1)	(1)
Head injury (*n* = 3)		(2)	(1)	(1)	(1)	(1)	(0)	(1)	(0)
Other injuries (fracture, contusion)	Fractures (total = 13)								
	Big toe fracture (*n* = 1)	(1)	(0)	(0)	(0)	(1)	(0)	(0)	(0)
	Thumb fracture (*n* = 2)	(2)	(2)	(2)	(0)	(1)	(1)	(0)	(0)
	Humorous fracture (*n* = 1)	(0)	(1)	(1)	(1)	(0)	(0)	(0)	(0)
	Ankle fracture (*n* = 4)	(2)	(4)	(4)	(1)	(1)	(0)	(2)	(0)
	Fibula fracture (*n* = 4)	(3)	(2)	(3)	(0)	(2)	(0)	(1)	(1)
	Tibia fracture (*n* = 1)	(1)	(1)	(0)	(0)	(1)	(0)	(0)	(0)
	Contusion (total = 3)								
	Shoulder (*n* = 1)	(1)	(1)	(1)	(0)	(1)	(0)	(0)	(0)
	Leg (*n* = 2)	(1)	(0)	(2)	(0)	(1)	(0)	(1)	(0)

The numbers between brackets represent the number of injuries for each category.

**Table 3:** Univariate logistic regression model for soccer injuries in Premier League and Division I teams.

	Injury Rate	β Coefficients	Nagelkerke R Square (R^2^)	OR	95%CI	*p*-Value
Age		0.137	10.2%	1.15	1.05–1.25	0.002
BMI		1.19	7.8%	1.21	1.06–1.38	0.003
Sex Male (*n* = 91) Females (*n* = 61) *	61.50% (*n* = 56) 19.70% (*n* = 12)	1.68	25.3%	5.39	2.11–13.75	<0.001
Dominant side Right (*n* = 116) Lift (*n* = 36) *	42.20% (*n* = 49) 52.80% (*n* = 19)	−0.58	15.7%	0.56	0.24–1.30	0.18
Team division Premier League (*n* = 74) Division I (*n* = 78) *	54.10% (*n* = 40) 35.90% (*n* = 28)	0.68	17.1%	1.98	0.96–4.09	0.065
Previous soccer injury Yes* (*n* = 19) No (*n* = 133)	84.2% (*n* = 16) 39.1% (*n* = 52)	2.12	20.1%	3.308	2.307–29.920	0.001
Playing surface Natural grass Synthetic surface *	56.88% (*n* = 62) 13.95% (*n* = 6)	2.40	34.6%	11.07	4.53–27.04	<0.001
Position Striker (*n* = 18) Defense (43) Goalkeeper (*n* = 13) Midfield (41) Side (25) *	50% (*n* = 9) 51.2% (*n* = 22) 61.5% (*n* = 8) 39% (*n* = 16) 48% (*n* = 12)	−0.295 −0.252 0.431 −0.714	16.77%	0.74 0.78 1.54 0.49	0.19–2.84 0.27–2.26 0.27–8.81 0.16–1.47	0.67 0.64 0.63 0.20

β: β coefficient, OR: odds ratio; CI: confidence interval, SD: standard deviation, * reference group for each predictor.

## Data Availability

All the relevant data of this study are available in the Department of Rehabilitation Sciences of Jordan University of Science and Technology, Irbid, Jordan. Data will be available upon a request sent to the corresponding author of this study.

## References

[1] Bizzini M., Junge A., Dvorak J. (2013). Implementation of the FIFA 11+ football warm up program: How to approach and convince the Football associations to invest in prevention. Br. J. Sports Med..

[2] Al-dawa D. List of Football Clubs in Jordan 2020, 18–19. https://en.wikipedia.org/wiki/List_of_football_clubs_in_England.

[3] Ekstrand J., Hagglund M., Walden M. (2009). Injury incidence and injury patterns in professional football: The UEFA injury study. Br. J. Sports Med..

[4] Walden M., Hägglund M., Ekstrand J. (2005). UEFA Champions League study: A prospective study of injuries in professional football during the 2001–2002 season. Br. J. Sports Med..

[5] Ekstrand J., Hägglund M., Kristenson K., Magnusson H., Waldén M. (2013). Fewer ligament injuries but no preventive effect on muscle injuries and severe injuries: An 11-year follow-up of the UEFA Champions League injury study. Br. J. Sports Med..

[6] Esquivel A.O., Bruder A., Ratkowiak K., Lemos S.E. (2015). Soccer-Related Injuries in Children and Adults Aged 5 to 49 Years in US Emergency Departments From 2000 to 2012. Sport. Health A Multidiscip. Approach.

[7] Hagglund M., Walden M., Ekstrand J. (2009). UEFA injury study—An injury audit of European Championships 2006 to 2008. Br. J. Sports Med..

[8] Meyers M.C. (2016). Incidence, Mechanisms, and Severity of Match-Related Collegiate Men’s Soccer Injuries on FieldTurf and Natural Grass Surfaces: A 6-Year Prospective Study. Am. J. Sports Med..

[9] Garcia P., Guodemar J., Ruiz M., Rodríguez-López E., Hervás-Pérez J.P. (2017). Injury Rate in Professional Soccer Players within the Community of Madrid: A Comparative, Epidemiological Cohort Study among the First, Second and Second B Divisions. J. Physiother. Phys. Rehabil..

[10] Reis G.F., Santos T.R.T., Lasmar R.C.P., Júnior O.O., Lopes R.F.F., Fonseca S.T. (2015). Sports injuries profile of a first division Brazilian soccer team: A descriptive cohort study. Braz. J. Phys. Ther..

[11] Onaka G.M., Gaspar-Jr J.J., Das Graças D., Barbosa F.S.S., Martinez P.F., De Oliveira-Junior S.A. (2017). Sports injuries in soccer according to tactical position: A retrospective survey. Fisioter. em Mov..

[12] del Pozo L.B., Pérez C.A., Benzanilla G.R., Fernández A.M., Villa T.F., Sánchez V.M. (2014). Influence of the soccer players’ professional status on the frequency and severity of injuries: A comparative pilot study. Apunt. Med. De L’esport.

[13] Ekstrand J., Gillquist J. (1983). Soccer injuries and their mechanisms. Med. Sci. Sports Exerc..

[14] Nielsen A.B., Yde J. (1989). Epidemiology and traumatology of injuries in soccer. Am. J. Sports Med..

[15] Pappas E., Shiyko M.P., Ford K.R., Myer G.D., Hewett T.E. (2016). Biomechanical Deficit Profiles Associated with ACL Injury Risk in Female Athletes. Med. Sci. Sports Exerc..

[16] Vlychou M., Hantes M., Michalitsis S., Tsezou A., Fezoulidis I.V., Malizos K. (2010). Chronic anterior cruciate ligament tears and associated meniscal and traumatic cartilage lesions: Evaluation with morphological sequences at 3.0 T. Skelet. Radiol..

[17] Beaulieu M.L., Lamontagne M., Xu L. (2008). Gender Differences in Time-Frequency EMG Analysis of Unanticipated Cutting Maneuvers. Med. Sci. Sports Exerc..

[18] Niyonsenga J., Phillips J. (2014). Factors associated with injuries among first-division Rwandan female soccer players. Afr. Health Sci..

[19] Arampatzis A., Degens H., Baltzopoulos V., Rittweger J. (2011). Why Do Older Sprinters Reach the Finish Line Later?. Exerc. Sport Sci. Rev..

[20] Hawkins R.D., Fuller C.W. (1999). A prospective epidemiological study of injuries in four English professional football clubs. Br. J. Sports Med..

[21] Di Salvo V., Baron R., Tschan H., Calderon Montero F.J., Bachl N., Pigozzi F. (2007). Performance Characteristics According to Playing Position in Elite Soccer. Int. J. Sports Med..

[22] Timpka T., Risto O., Björmsjö M. (2007). Boys soccer league injuries: A community-based study of time-loss from sports participation and long-term sequelae. Eur. J. Public Health.

[23] Ekstrand J., Timpka T., Hägglund M., Karlsson J. (2006). Risk of injury in elite football played on artificial turf versus natural grass: A prospective two-cohort study. Commentary. Br. J. Sports Med..

[24] Hershman E.B., Anderson R., Bergfeld J.A., Bradley J.P., Coughlin M.J., Johnson R.J., Spindler K.P., Wojtys E., Powell J.W., Collins J.T. (2012). An Analysis of Specific Lower Extremity Injury Rates on Grass and FieldTurf Playing Surfaces in National Football League Games. Am. J. Sports Med..

[25] Hagglund M., Waldén M., Ekstrand J. (2006). Previous injury as a risk factor for injury in elite football: A prospective study over two consecutive seasons. Br. J. Sports Med..

[26] Walden M., Hägglund M., Ekstrand J. (2006). High risk of new knee injury in elite footballers with previous anterior cruciate ligament injury. Br. J. Sports Med..

[27] Fuller C.W., Molloy M.G., Bagate C., Bahr R., Brooks J.H.M., Donson H., Kemp S.P.T., McCrory P., McIntosh A.S., Meeuwisse W.H. (2007). Consensus statement on injury definitions and data collection procedures for studies of injuries in rugby union. Br. J. Sports Med..

[28] Kilic O., Aoki H., Goedhart E., Hägglund M., Kerkhoffs G.M.M.J., Kuijer P.P.F., Waldén M., Gouttebarge V. (2017). Severe musculoskeletal time-loss injuries and symptoms of common mental disorders in professional soccer: A longitudinal analysis of 12-month follow-up data. Knee Surg. Sports Traumatol. Arthrosc..

[29] Gouttebarge V., Aoki H., Ekstrand J., Verhagen E.A.L.M., Kerkhoffs G.M.M.J. (2015). Are severe musculoskeletal injuries associated with symptoms of common mental disorders among male European professional footballers?. Knee Surg. Sport. Traumatol. Arthrosc..

[30] Dennis J., Dawson B., Heasman J., Rogalski B., Robey E. (2016). Sleep patterns and injury occurrence in elite Australian footballers. J. Sci. Med. Sport.

[31] Morgan B.E., Oberlander M.A. (2001). An Examination of Injuries in Major League Soccer. Am. J. Sports Med..

[32] Stubbe J.H., van Beijsterveldt A.-M.M.C., van der Knaap S., Stege J., Verhagen E.A., van Mechelen W., Backx F.J.G. (2015). Injuries in Professional Male Soccer Players in the Netherlands: A Prospective Cohort Study. J. Athl. Train..

[33] Dvorak J., Junge A., Grimm K., Kirkendall D. (2007). Medical report from the 2006 FIFA World Cup Germany. Br. J. Sports Med..

[34] Read P.J., Oliver J.L., De Ste Croix M.B., Myer G.D., Lloyd R.S. (2018). An audit of injuries in six english professional soccer academies. J. Sports Sci..

[35] Richmond S.A., Nettel-Aguirre A., Doyle-Baker P.K., Macpherson A., Emery C.A. (2016). Examining Measures of Weight as Risk Factors for Sport-Related Injury in Adolescents. J. Sports Med..

[36] Larruskain J., Lekue J.A., Diaz N., Odriozola A., Gil S.M. (2017). A comparison of injuries in elite male and female football players: A five-season prospective study. Scand. J. Med. Sci. Sports.

[37] Del Coso J., Herrero H., Salinero J.J. (2016). Injuries in Spanish female soccer players. J. Sport Health Sci..

[38] Roos K.G., Wasserman E.B., Dalton S.L., Gray A., Djoko A., Dompier T.P., Kerr Z.Y. (2017). Epidemiology of 3825 injuries sustained in six seasons of National Collegiate Athletic Association men’s and women’s soccer (2009/2010–2014/2015). Br. J. Sports Med..

[39] Meyers M.C. (2010). Incidence, Mechanisms, and Severity of Game-Related College Football Injuries on FieldTurf versus Natural Grass: A 3-year prospective study. Am. J. Sports Med..

[40] Vorslenbosch C.C.M., Staal J.B., Kolenburg L., Meijer K. Football injuries on artificial grass and natural grass: Comparative incidence and location. Sport en Geneeskd..

[41] Aoki H., Kohno T., Fujiya H., Kato H., Yatabe K., Morikawa T., Seki J. (2010). Incidence of Injury Among Adolescent Soccer Players: A Comparative Study of Artificial and Natural Grass Turfs. Clin. J. Sport Med..

[42] Meyers M.C., Barnhill B.S. (2004). Incidence, Causes, and Severity of High School Football Injuries on FieldTurf versus Natural Grass: A 5-year prospective study. Am. J. Sport. Med..

[43] Williams S., Hume P.A., Kara S. (2011). A Review of Football Injuries on Third and Fourth Generation Artificial Turfs Compared with Natural Turf. Sports Med..

[44] Ekstrand J., Gillquist J. (1982). The frequency of muscle tightness and injuries in soccer players. Am. J. Sports Med..

[45] Brophy R., Silvers H.J., Gonzales T., Mandelbaum B.R. (2010). Gender influences: The role of leg dominance in ACL injury among soccer players. Br. J. Sports Med..

